# Distinct retinal oxygenation patterns in acute zonal occult outer retinopathy assessed by multimodal imaging

**DOI:** 10.3389/fnimg.2026.1902215

**Published:** 2026-09-09

**Authors:** Scott Tschuppert, Christophe Valmaggia, Margarita G. Todorova

**Affiliations:** 1 Department of Ophthalmology, HOCH Health Ostschweiz, St. Gallen, Switzerland; 2 Department of Ophthalmology, University of Zurich, Zurich, Switzerland

**Keywords:** acute zonal occult outer retinopathy, AZOOR, ellipsoid zone, mfERG, multimodal imaging, retinal metabolism, retinal oximetry, retinitis pigmentosa

## Abstract

**Purpose:**

Acute zonal occult outer retinopathy (AZOOR) is a rare outer retinal disorder that may mimic inherited retinal dystrophy. This study revises a retrospective AZOOR case series by integrating retinal oximetry as a metabolic imaging biomarker and comparing the oximetry findings with retinitis pigmentosa (RP) and healthy control data.

**Methods:**

Seven consecutive patients diagnosed with AZOOR at HOCH Health Ostschweiz in St. Gallen, Switzerland were retrospectively reviewed. Multimodal assessment included best-corrected visual acuity, fundus examination, spectral-domain optical coherence tomography (SD-OCT), fundus autofluorescence (FAF), near-infrared reflectance (NIR-R), fluorescein angiography, visual field testing, multifocal electroretinography (mfERG), full-field electroretinography (ffERG), laboratory work-up, and magnetic resonance imaging. Retinal vessel oxygen saturation data (arteriolar: A-SO_2_, venular: V-SO_2_ and their difference: A-V SO_2_) were available in four AZOOR patients and compared with an existing institutional retinal oximetry dataset containing RP patients and healthy controls. Retinal oxygen saturation and vessel diameter were assessed using Oxymap T1 dual-wavelength retinal oximetry in a predefined peripapillary measurement area. Because of the limited AZOOR sample, all comparative analyses were exploratory.

**Results:**

The AZOOR cohort comprised seven patients (5 female, 2 male; median age 28 years, range 20–53). Six patients presented unilaterally; one patient had congenital aniridia bilaterally but AZOOR was clinically assigned to one presenting eye. Ellipsoid-zone disruption on SD-OCT was present in all affected eyes. MfERG demonstrated macular dysfunction in six of seven patients, whereas ffERG was abnormal in five of seven patients or showed normal full-field responses. Complete retinal oximetry data were available for eight eye/image measurements from four patients. Kruskal–Wallis testing across controls, RP, and AZOOR showed significant intergroup differences for A-SO_2_ (*H* = 10.406, df = 2, *p* = 0.005) and A-V SO_2_ (*H* = 9.538, df = 2, *p* = 0.008), but not for V-SO_2_ (*H* = 1.002, df = 2, *p* = 0.606). Exploratory pairwise Mann–Whitney *U* tests showed lower A-SO_2_ and lower A-V SO_2_ in AZOOR compared with controls and RP, whereas V-SO_2_ did not differ significantly.

**Conclusion:**

Retinal oximetry added a metabolic dimension to multimodal AZOOR assessment. In this small exploratory cohort, AZOOR showed reduced arterial saturation and reduced arteriovenous oxygen difference compared with controls and RP, whereas venous saturation was not altered. This distinct metabolic AZOOR profile indicates reduced oxygen delivery resulting in altered oxygen use contrary to the known RP pattern of increased oxygen saturation and reduced oxygen use associated with photoreceptor degeneration. These findings should be interpreted cautiously but support further prospective evaluation of retinal oximetry as a complementary imaging biomarker in AZOOR and other outer retinal disorders.

## Introduction

Acute zonal occult outer retinopathy (AZOOR) was introduced by Gass as a syndrome characterized by acute loss of one or more zones of outer retinal function, photopsias or scotomas, minimal funduscopic changes, visual-field loss, and electroretinographic abnormalities (Gass, 1993; Gass et al., 2002). Despite more than three decades of clinical experience, AZOOR remains incompletely understood. Its rarity, heterogeneous presentation, overlap with white-dot syndromes or inherited retinal dystrophy, and sometimes subtle fundus appearance contribute to diagnostic delay and uncertainty regarding treatment.

Modern multimodal imaging has substantially refined the clinical diagnosis. Mrejen and colleagues proposed a multimodal imaging-based classification that emphasized trizonal patterns, ellipsoid-zone disruption on SD-OCT, fundus autofluorescence changes, and near-infrared reflectance abnormalities (Mrejen et al., 2014). Subsequent reviews and reports support the importance of combining structural imaging, functional testing, and electrophysiology rather than relying on fundus appearance alone (Guijarro et al., 2022; Mrejen et al., 2014; Pellegrini et al., 2021). Adaptive optics imaging has further expanded the ability to visualize photoreceptor-level alterations (Xu et al., 2022).

The pathophysiology of AZOOR is unresolved. Proposed mechanisms include immune-mediated injury, post-infectious or viral triggers, anti-retinal antibodies, and degenerative secondary remodeling (Gass, 1993; Gass et al., 2002; Qian et al., 2017; Zeng et al., 2019). Treatment remains controversial. Systemic corticosteroids, antiviral medication, local corticosteroids, and longer-term immunosuppression have been reported, but no controlled trial has established a standard regimen (Barnes et al., 2018; Chen et al., 2015; Jampol and Becker, 2003; Saito et al., 2015). Consequently, objective biomarkers that capture disease activity, tissue injury, and retinal metabolism could be useful in both clinical monitoring and future interventional studies.

Retinal oximetry is a non-invasive imaging approach that estimates oxygen saturation in retinal vessels by differential light absorption of oxygenated and deoxygenated haemoglobin (Stefánsson et al., 2019). This novel non-contact imaging allows evaluation of retinal metabolic function. In photoreceptor degenerations such as retinitis pigmentosa (RP), retinal oximetry has shown altered oxygen saturation, vessel narrowing, and changes consistent with metabolic demand (reduced retinal oxygen use) as photoreceptor metabolism declines (Bojinova et al., 2018; Eysteinsson et al., 2014; Todorova et al., 2016; Türksever et al., 2014). Because AZOOR primarily affects the outer retina, retinal oximetry may provide complementary information on metabolic consequences of focal photoreceptor dysfunction that are not captured by conventional structural imaging alone.

The purpose of this revised study was therefore to update a retrospective AZOOR case series by integrating retinal oximetry, comparing the available AZOOR oximetry data with RP and healthy control cohorts, and positioning retinal oximetry as a potential metabolic imaging biomarker within multimodal assessment of AZOOR.

## Materials and methods

### Study design and patients

This was a retrospective observational case series of consecutive patients diagnosed with AZOOR between October 2005 and October 2025 at the Department of Ophthalmology, HOCH Health Ostschweiz, Cantonal Hospital St. Gallen, Switzerland. The diagnosis was based on the original Gass criteria and contemporary multimodal imaging features: acute subjective visual symptoms, focal or zonal outer retinal dysfunction, minimal or mild funduscopic findings at presentation, visual-field or electrophysiologic abnormalities, and corresponding outer retinal abnormalities on SD-OCT, FAF, or NIR-R (Gass, 1993; Gass et al., 2002; Mrejen et al., 2014). Seven AZOOR patients were included.

The study was conducted in accordance with the Declaration of Helsinki and approved by the responsible ethics committee trial number EKNZ BASEC 2017-00937.

### Clinical and multimodal imaging assessment

Clinical data included age, sex, symptoms, time to diagnosis, primarily affected eye, BCVA at presentation and last follow-up, personal history, complications, laboratory findings, MRI findings, and treatment. Imaging and functional assessment included color fundus photography, SD-OCT, FAF, NIR-R, fluorescein angiography and/or indocyanine green angiography when clinically indicated, Goldmann or Octopus perimetry, mfERG, and ffERG according to ISCEV recommendations available at the time of testing (Hood et al., 2012; McCulloch et al., 2015).

### Retinal oximetry

Retinal oximetry was available in four AZOOR patients. The remaining patients had been diagnosed and completed their clinical follow-up before retinal oximetry was introduced into routine clinical imaging at our institution. Consequently, retinal oximetry data were unavailable for these patients because the examination had not been performed, and not because of inadequate image quality. Retinal vessel oximetry was performed using the Oxymap T1 spectrophotometric retinal oximeter (Oxymap ehf., Reykjavik, Iceland) mounted on a Topcon TRC-50DX fundus camera (Topcon Corporation, Tokyo, Japan), with image analysis performed using Oxymap Analyzer software (version 2.5.2; Oxymap ehf., Reykjavik, Iceland). Optic-disc-centred retinal images were acquired simultaneously at two wavelength ranges: an oxygen-insensitive isosbestic channel at 586 ± 10 nm and an oxygen-sensitive non-isosbestic channel at 605 ± 10 nm. The software differentiated oxygenated from deoxygenated haemoglobin on the basis of their wavelength-dependent optical density characteristics.

For analysis, two concentric circles with radii of two and three optic-disc diameters were positioned around the optic disc. The peripapillary region between these circles defined the area of interest. All major retinal arterioles and venules passing through this region were selected manually. The Oxymap Analyzer software (version 2.5.2; vessel locator v2) calculated the optical density ratio between the two wavelength images and estimated oxygen saturation for each selected vessel segment. Global mean arteriolar oxygen saturation (A-SO2) and venular oxygen saturation (V-SO2) were calculated for each analyzed eye/image, and the arterio-venular oxygen saturation difference (A-V SO2) was calculated as A-SO2 minus V-SO2. The corresponding arteriolar and venular vessel diameters were also recorded. The mean saturation value of all eligible arteriolar segments and all eligible venular segments within the predefined peripapillary measurement area was calculated for each eye/image.

**Figure 1:**
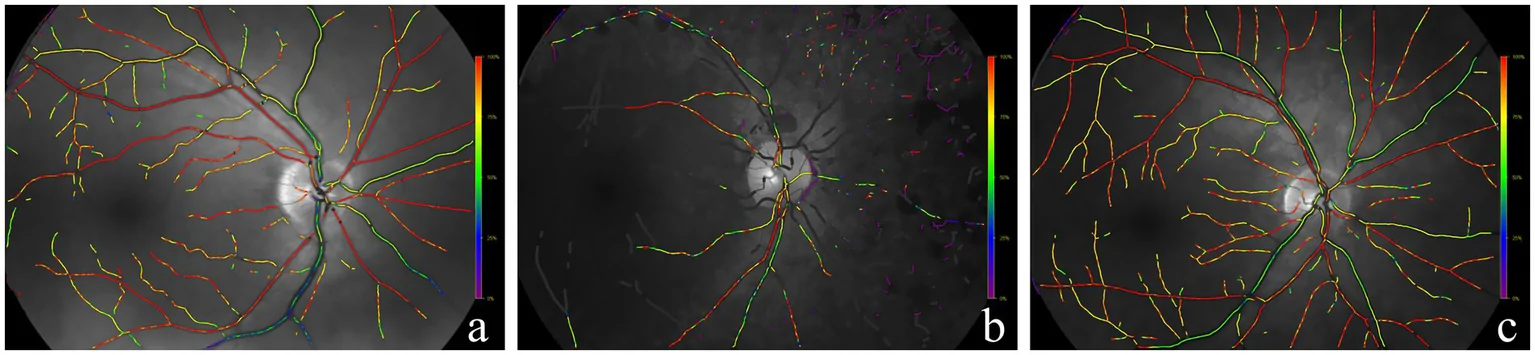
Representative retinal oximetry images in **(a)** healthy control, **(b)** retinitis pigmentosa (RP), and **(c)** acute zonal occult outer retinopathy (AZOOR), matching the order used throughout the comparative analyses. Pseudocolor overlays indicate retinal vessel oxygen saturation according to the scale bar; warmer colors indicate higher measured saturation and cooler colors indicate lower measured saturation.

For each eye, the image with the best available centration, focus, illumination, and vessel delineation was selected for analysis. Images or vessel segments with inadequate focus, centration, illumination, incomplete visualization of the predefined peripapillary measurement area, or uncertain vessel classification were excluded from quantitative analysis. All retinal oximetry images included in the present study fulfilled these predefined quality criteria. Because retinal oximetry was retrospectively available only in a subset of the AZOOR cohort, the measurements were analyzed as exploratory eye/image-level data. Representative retinal oximetry images from a healthy control, an eye with retinitis pigmentosa, and an eye with AZOOR are shown in Figure 1.

### Comparative RP and control cohort

A separate dataset containing retinal oximetry in RP and healthy control participants was used for exploratory comparison. Age-matched healthy controls and patients with clinically and electrodiagnostically confirmed retinitis pigmentosa originated from an existing institutional retinal oximetry database acquired using the identical imaging protocol and device. The RP and healthy control groups were age-matched to each other within the original institutional dataset; the AZOOR patients were not individually age-matched to either comparative group. The RP cohort consisted of patients with clinically and electrophysiologically confirmed retinitis pigmentosa. A formal standardized RP severity classification was not available in the source dataset. The RP and healthy control cohorts were examined at the Department of Ophthalmology, HOCH Health Ostschweiz, using the same retinal oximetry device, acquisition protocol, and image analysis strategy as the present AZOOR cohort. As all measurements were performed at the same institution using the same retinal oximetry system, no changes in calibration procedures occurred between acquisition of the comparative RP/control dataset and the AZOOR measurements. The RP and healthy control measurements were acquired between September 2019 and August 2021 using the same predefined image-quality criteria as applied for the AZOOR retinal oximetry analysis. The comparative dataset comprised 22 eyes from 15 healthy control participants (9 men, 6 women; mean age 46.8 ± 15.7 years) and 42 eyes from 21 patients with retinitis pigmentosa (10 men, 11 women; mean age 49.8 ± 15.2 years). Healthy control participants had no history of retinal disease, glaucoma, diabetes mellitus, retinal vascular disease, or other ocular disorders known to affect retinal oxygen saturation. The dataset did not include AZOOR patients. Eye-level arterial saturation, venous saturation, arteriovenous difference, and age were extracted. These data were combined with the AZOOR eye/image-level measurements for comparative analysis.

### Statistical analysis

Descriptive statistics are reported as mean ± standard deviation and median with interquartile range where appropriate. Because the AZOOR oximetry sample was small, group comparisons were exploratory and interpreted as hypothesis-generating. Because both eyes from the same participant were included whenever available, observations were not statistically independent. Therefore, statistical analyses should be interpreted as exploratory and hypothesis-generating rather than confirmatory. Accordingly, *p*-values are presented descriptively and should be interpreted as exploratory rather than confirmatory evidence. Primary group comparisons across controls, RP, and AZOOR were performed with the Kruskal–Wallis *H* test. Pairwise post-hoc comparisons were performed with Mann–Whitney *U* tests. Pairwise analyses were considered exploratory and were not adjusted for multiple comparisons because of the small sample size and hypothesis-generating study design. Oxygen saturation values exceeding 100% were retained in the primary analysis and were not capped, because such values are recognized in retinal oximetry of advanced retinal degeneration and are generally interpreted as reflecting device calibration characteristics together with disease-related changes in retinal vessel caliber and retinal optical properties rather than true supraphysiological oxygen saturation. Analyses were recalculated in IBM SPSS Statistics version 25.0.0.2.

## Results

### Clinical cohort

The updated AZOOR cohort included seven patients: five women and two men. Median age at presentation was 28 years (range 20–53 years). The presenting symptoms included scotoma, photopsia, blurred vision, visual acuity reduction, color desaturation, and visual-field defects. Time to diagnosis ranged from 8 to 83 days among patients with available data. Follow-up ranged from 0 to 120 months. One patient developed fellow-eye involvement during follow-up and one experienced recurrence. The newest patient had known bilateral aniridia and presented with unilateral AZOOR-compatible outer retinal dysfunction. Detailed demographic and clinical characteristics of the AZOOR cohort are summarized in Table 1.

**Table 1:** Clinical characteristics of the AZOOR cohort.

No.	Age	Sex	Symptoms	Eye	Diagnostic delay (days)	Final BCVA	Follow-up (months)	Key history/complications
1	24	F	Visual acuity reduction, visual field defect	OD	8	1.0	57	Ovarian teratoma; sinus thrombosis
2	22	F	Photopsia, visual field defect	OS	20	1.20	34	Recurrence
3	43	F	Blurred vision	OS	60	0.02	120	Psoriasis; fellow-eye involvement
4	28	M	Central scotoma	OD	83	0.20	38	Occasional smoker
5	53	F	Paracentral scotoma	OS	n/a	n/a	0	Preceding flu-like illness
6	35	M	Visual acuity reduction, color desaturation	OS	17	0.90	5	Thoracic herpes zoster later
7	20	F	Visual acuity reduction	OS	180	0.16	50	Bilateral congenital aniridia

BCVA, best-corrected visual acuity.

### Structural and functional findings

**Figure 2:**
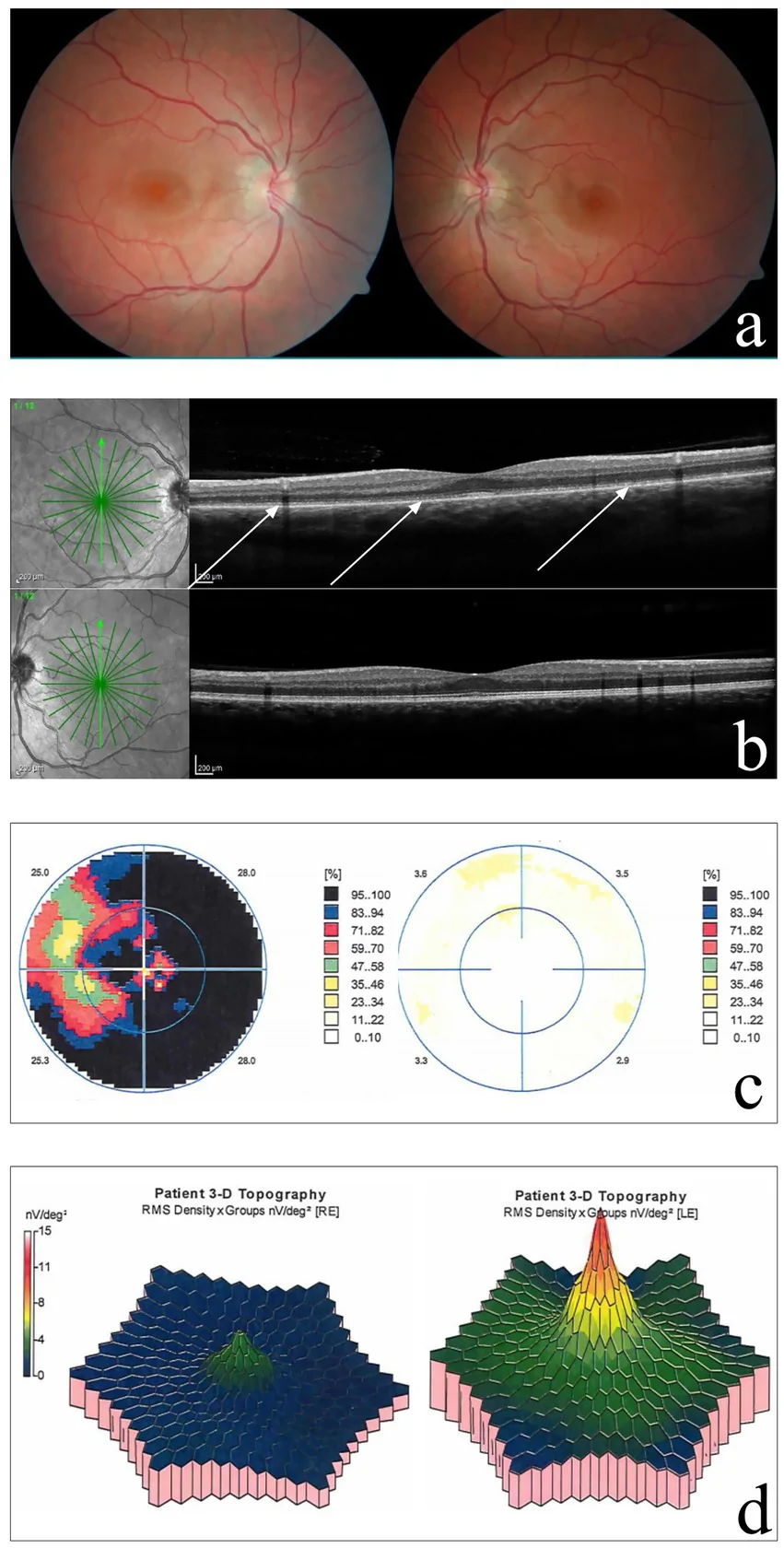
Patient 1, a 24-year-old female: first presentation. **(a)** Fundus photography, unremarkable. **(b)** SD-OCT of the right eye showing alterations in the ellipsoid zone (arrows) and a mottled appearance on NIR-R. **(c)** Octopus perimetry showing major defects in the right eye. **(d)** mfERG showing dysfunction of the whole macula in the right eye.

**Figure 3:**
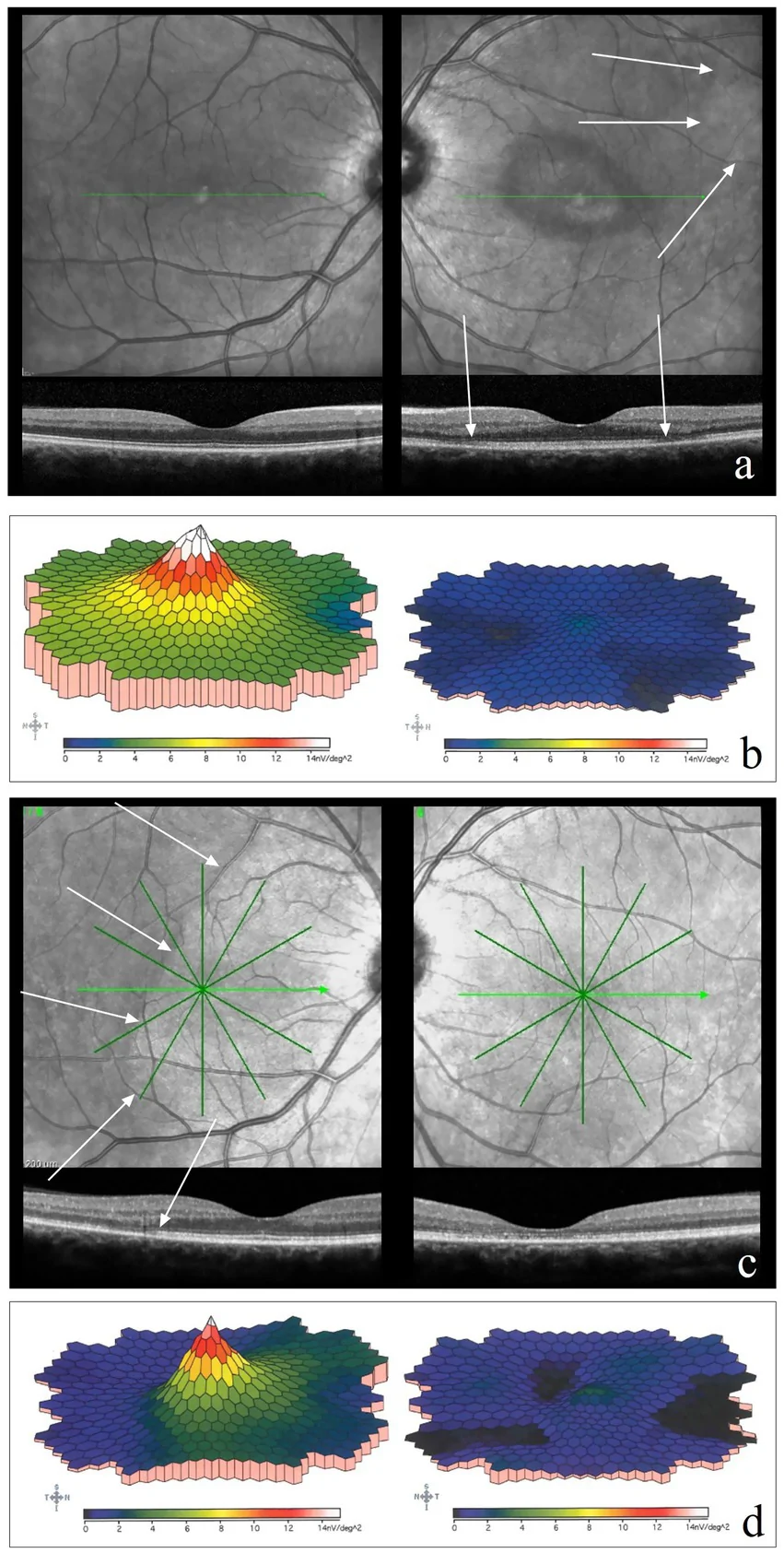
Patient 3, a 43-year-old female. **(a, b)** First presentation. **(a)** NIR-R showing a mottled appearance (arrows) and central ellipsoid-zone alterations; SD-OCT showing alterations in the ellipsoid zone (arrows). **(b)** mfERG showing severe dysfunction of the whole macula in the left eye. **(c, d)** Presentation of the newly affected fellow eye during follow-up. **(c)** NIR-R showing a demarcation line (arrows) of the affected retina, correlating with alterations of the ellipsoid zone on SD-OCT (arrows). **(d)** mfERG showing corresponding demarcation of the affected retinal area.

Fundus examination was normal or only subtly abnormal in most patients. SD-OCT showed ellipsoid-zone irregularity or loss in all affected eyes. FAF and NIR-R demonstrated hyperautofluorescent or trizonal/mottled abnormalities in the affected retina when available. MfERG revealed macular dysfunction in six of seven patients, whereas full-field ERG ranged from normal to reduced scotopic and/or photopic responses. Visual-field testing, when interpretable, showed enlarged blind spot, central or paracentral scotoma, or field defects corresponding to multimodal imaging abnormalities. Structural imaging and electrophysiological findings are presented in Tables 2, 3. Representative multimodal imaging examples demonstrating characteristic AZOOR findings are shown in Figures 2, 3.

**Table 2:** Structural imaging findings in the AZOOR cohort.

No.	SD-OCT	FAF	NIR-R
1	Irregular ellipsoid zone extending from optic disc	Patchy hyperautofluorescence	Trizonal pattern
2	Inferotemporal ellipsoid zone disruption	Discrete trizonal pattern	Trizonal pattern
3	Irregular foveal ellipsoid zone	Reduced xanthophyll masking	Foveal trizonal pattern
4	Irregular ellipsoid zone extending from optic disc	Non-interpretable	Trizonal pattern
5	Inferonasal foveal ellipsoid zone irregularity	Not performed	Bizonal pattern
6	Attenuated ellipsoid zone toward optic disc	Patchy hyperautofluorescence	Patchy hypoautofluorescence
7	Irregular ellipsoid zone; fovea plana	Patchy hyperautofluorescence	Trizonal pattern

SD-OCT, spectral-domain optical coherence tomography; FAF, fundus autofluorescence; NIR-R, near-infrared reflectance.

**Table 3:** Electrophysiological findings in the AZOOR cohort.

No.	mfERG	ffERG
1	Global macular dysfunction	Scotopic response reduced by 30–35%; cone response reduced by 50%
2	Inferotemporal macular dysfunction	Normal rod response; cone response reduced by 25%
3	Global macular dysfunction; fellow-eye involvement	Rod response normal; cone response reduced OD and extinguished OS
4	Global macular dysfunction, predominantly nasal	Rod and cone responses reduced by ~20%
5	Normal	Not performed
6	Global macular dysfunction	Normal
7	Global macular dysfunction, predominantly temporal	Scotopic and photopic responses within normal limits

mfERG, multifocal electroretinography; ffERG, full-field electroretinography.

### Retinal oximetry in AZOOR

Retinal oximetry was available in four patients (Table 4). Retinal oximetry was performed during routine clinical follow-up whenever the technique became available and was therefore not standardized to symptom onset or disease stage. The interval from symptom onset to retinal oximetry was approximately 15 days, 35 months, 43 months, and 120 months for the four patients with available retinal oximetry data. Accordingly, retinal oximetry was performed during the acute stage in one patient and during the chronic stage in the remaining three patients. Eight complete eye/image measurements with arterial and venous measurements were included in the primary statistical analysis; one additional image contained only arterial segments and was therefore excluded from group comparisons requiring complete arterial, venous, and arteriovenous difference data. Across the eight complete AZOOR oximetry summaries, mean A-SO_2_ was 95.2 ± 2.4%, mean V-SO_2_ was 67.3 ± 6.0%, and mean A-V SO_2_ was 27.9 ± 4.4%. Individual AZOOR retinal oximetry measurements are shown in Table 4.

**Table 4:** Retinal oximetry measurements in AZOOR eyes.

Patient ID	Eye	Status	Time from symptom onset to oximetry	A-SO_2_ (%)	V-SO_2_ (%)	A-V SO_2_ (%)
1	OD	Affected	35 months	98.8	76.26	22.53
1	OS	Fellow	35 months	98.19	72.49	25.7
2	OS	Affected	43 months	93.34	65.59	27.75
2	OD	Fellow	43 months	93.96	67.57	26.39
3	OS	Affected	120 months	93.66	62.45	31.21
3	OD	Fellow	120 months	92.62	67.12	25.5
7	OS	Affected	15 days	96.94	69.75	27.18
7	OD	Fellow	15 days	93.69	56.88	36.81

A-SO_2_, arterial oxygen saturation; V-SO_2_, venous oxygen saturation; A-V SO_2_, arteriovenous oxygen saturation difference.

A sensitivity analysis excluding the patient with bilateral congenital aniridia (Patient 7) yielded comparable exploratory retinal oximetry findings. The direction of all observed retinal oxygen saturation differences remained unchanged, with lower arterial oxygen saturation (A-SO₂), persistently non-significant differences in venous oxygen saturation (V-SO₂), and lower arteriovenous oxygen saturation difference (A–V SO₂) compared with healthy controls.

### Comparative oximetry analysis

**Figure 4:**
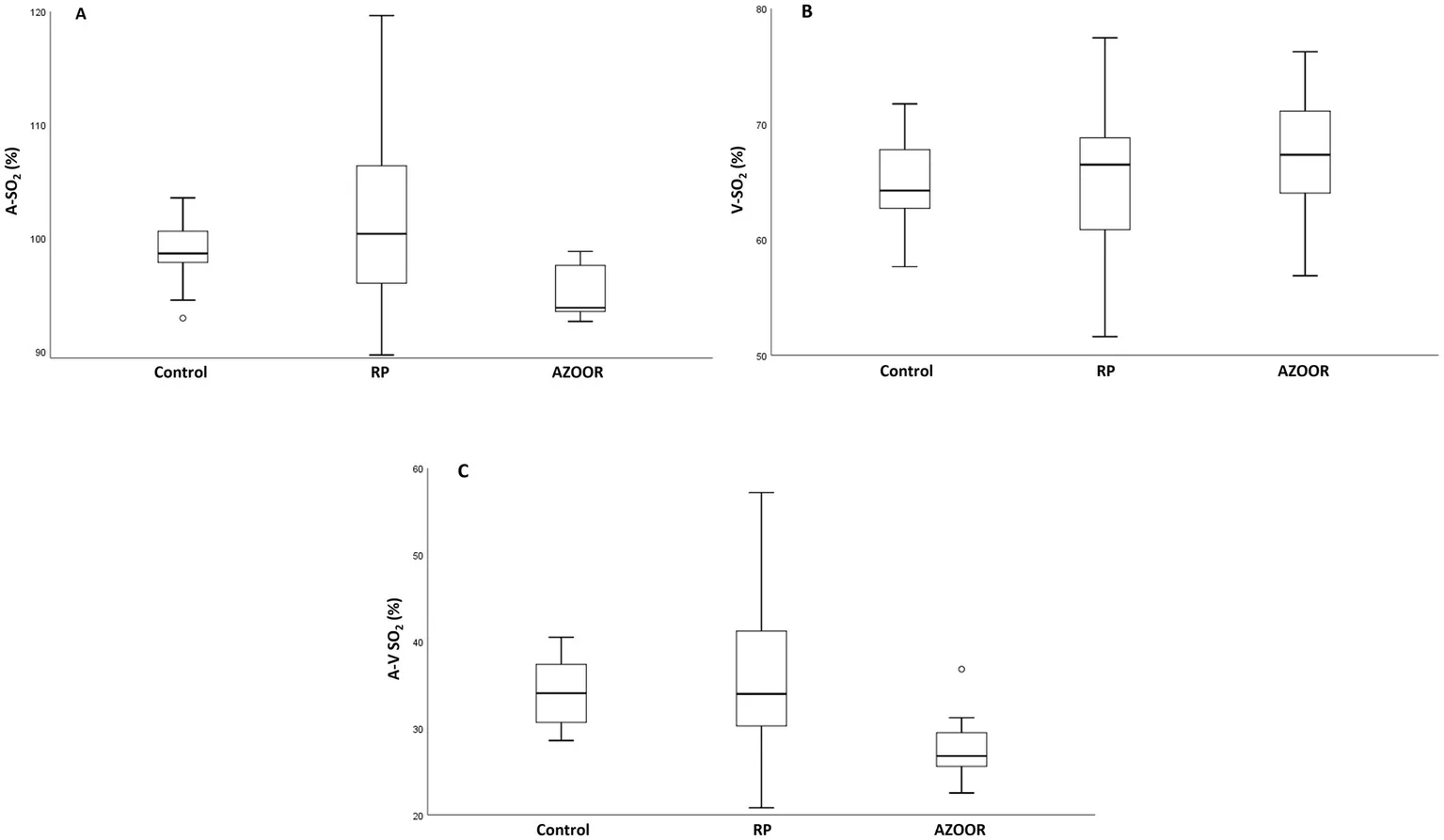
Retinal oxygen saturation measurements in healthy controls, retinitis pigmentosa (RP), and acute zonal occult outer retinopathy (AZOOR). **(A)** Arterial oxygen saturation (A-SO_2_) **(B)** Venous oxygen saturation (V-SO_2_) and **(C)** Arteriovenous oxygen saturation difference (A-V SO_2_). Boxplots represent median and interquartile range; whiskers indicate minimum and maximum non-outlier values. Individual outliers are displayed separately.

The comparative dataset included 22 control eyes/images, 42 RP eyes/images, and eight complete AZOOR oximetry summaries (total *n* = 72). Descriptive retinal oximetry values for all groups are summarized in Table 5. Kruskal–Wallis analysis demonstrated significant intergroup differences for A-SO_2_ (*H* = 10.406, df = 2, *p* = 0.005) and A-V SO_2_ (*H* = 9.538, df = 2, *p* = 0.008), whereas V-SO_2_ did not differ significantly between groups (*H* = 1.002, df = 2, *p* = 0.606). Overall, group comparisons are presented in Table 6. Exploratory pairwise Mann–Whitney analyses revealed significantly lower A-SO_2_ and A-V SO_2_ in AZOOR eyes/images compared with both healthy controls and RP. Detailed pairwise comparisons are shown in Table 7. RP and controls did not differ significantly in the present dataset. V-SO_2_ showed no significant pairwise group differences. The distribution of A-SO_2_, V-SO_2_, and A-V SO_2_ across controls, RP, and AZOOR is illustrated in Figure 4.

**Table 5:** Comparative retinal oximetry summary.

Group	*N*	A-SO_2_ (%)	V-SO_2_ (%)	A-V SO_2_ (%)
Healthy Control	22	99.1 ± 2.6	64.9 ± 3.9	34.2 ± 4.0
RP	42	101.3 ± 6.7	65.5 ± 6.2	35.8 ± 8.2
AZOOR	8	95.2 ± 2.4	67.3 ± 6.0	27.9 ± 4.4

RP, retinitis pigmentosa.

**Table 6:** Overall Kruskal–Wallis comparisons across controls, RP, and AZOOR.

Variable	Kruskal–Wallis *H*	df	*p*
A-SO_2_	10.406	2	0.005
V-SO_2_	1.002	2	0.606
A-V SO_2_	9.538	2	0.008

**Table 7:** Exploratory pairwise Mann–Whitney *U* comparisons.

Variable	Comparison	Mann–Whitney *U*	*Z*	*p*	Interpretation
A-SO_2_	AZOOR vs. Control	25.0	−2.955	0.003	AZOOR lower
A-SO_2_	AZOOR vs. RP	61.0	−2.832	0.005	AZOOR lower
V-SO_2_	AZOOR vs. Control	67.0	−0.985	0.325	
V-SO_2_	AZOOR vs. RP	137.0	−0.82	0.412	
A-V SO_2_	AZOOR vs. Control	22.0	−3.095	0.002	AZOOR lower
A-V SO_2_	AZOOR vs. RP	63.0	−2.779	0.005	AZOOR lower

Pairwise analyses were exploratory and were not corrected for multiple testing.

## Discussion

### Established AZOOR hallmarks

The clinical and imaging phenotype in this cohort was consistent with the established hallmarks of AZOOR. Patients presented with acute or subacute visual symptoms, including scotoma, photopsia, visual acuity reduction, color desaturation, and visual-field defects, while funduscopic findings were absent or subtle in most cases. Across the cohort, SD-OCT demonstrated ellipsoid-zone disturbance in affected eyes, FAF and NIR-R images showed zonal or mottled abnormalities when available, and mfERG detected macular dysfunction in most patients. Full-field ERG revealed reduced photopic and 30 Hz flicker responses. These findings support the current concept that AZOOR is best recognized by integrating structural imaging with functional testing rather than by fundus appearance alone (Gass, 1993; Gass et al., 2002; Mrejen et al., 2014; Pellegrini et al., 2021).

### Contribution of retinal oximetry

Retinal oximetry added a complementary metabolic layer to this multimodal phenotype. Conventional AZOOR imaging identifies the location and extent of outer retinal disruption, whereas retinal oximetry provides vessel-based information on retinal oxygenation and thus on retinal metabolic function. In the present dataset, AZOOR eyes/images showed significantly lower A-SO_2_ and A-V SO_2_ compared with both controls and RP, while V-SO_2_ was not significantly different. This pattern suggests that retinal oximetry may capture disease-associated metabolic or vascular alterations that are not fully reflected by SD-OCT, FAF, NIR-R, ffERG or mfERG alone (Hardarson, 2013; Olafsdottir et al., 2014; Stefánsson et al., 2019).

### Interpretation of the RP comparison

In healthy retinal circulation, arteriolar oxygen saturation is higher than venular oxygen saturation, and the arteriovenous oxygen saturation difference reflects the decline in oxygen saturation across the retinal circulation. This difference is used as an indirect marker of retinal oxygen use but does not directly measure retinal blood flow, perfusion, or absolute tissue oxygen consumption. The comparison with RP is informative because RP is a chronic progressive generalized photoreceptor degeneration with a well-described retinal metabolic pattern. In RP, degeneration of photoreceptors, particularly rods with high oxygen demand, reduces oxygen consumption and oxygen use. Previous studies from retinal oximetry cohorts have reported increased retinal vessel oxygen saturation, vascular narrowing, and reduced oxygen use patterns in RP, and these observations are consistent with the present RP comparison group (Bojinova et al., 2018; Eysteinsson et al., 2014; Todorova et al., 2016; Türksever et al., 2014). Values exceeding 100% in RP should not be interpreted as true physiological suprasaturation; they are recognized measurement phenomena related to altered vessel caliber, retinal thinning, and tissue optical properties (Bojinova et al., 2018; Eysteinsson et al., 2014; Todorova et al., 2016; Türksever et al., 2014). Therefore, these raw values were retained rather than capped, because truncation would artificially compress variance and bias group comparisons.

### Metabolic pattern in AZOOR and inflammatory-occlusive considerations

AZOOR differed from both the healthy control and RP patterns. Compared with healthy controls, the AZOOR measurements showed lower A-SO₂ and lower A–V SO₂, whereas V-SO₂ was not significantly different. This pattern cannot be explained by reduced photoreceptor oxygen use alone, because reduced oxygen use, as observed in RP, would generally be expected to increase rather than decrease retinal vessel oxygen saturation.

The lower measured A-SO₂ in AZOOR indicates lower arteriolar oxygen saturation at the retinal measurement site, but it does not by itself demonstrate reduced blood flow or reduced perfusion. Retinal oximetry measures haemoglobin oxygen saturation rather than volumetric blood flow, and the present data therefore cannot determine why A-SO₂ was lower. Potential explanations may include local vascular dysregulation, inflammation-associated vascular changes, disease-related optical characteristics, or other disease-associated factors affecting retinal oxygen saturation measurements; however, these possibilities remain hypothetical.

Retinal oximetry alone cannot prove occlusion or reduced perfusion, because it measures vessel oxygen saturation rather than blood flow. In inflammatory retinal diseases, such as VKH uveitis, retinal oxygen metabolism and blood-flow parameters can be altered during active disease, and in Behcet uveitis, posterior involvement is characterized by active or occlusive retinal vasculitis detectable by angiography and OCTA (Abu El-Asrar et al., 2019; Khairallah et al., 2017; Khairallah et al., 2012; Tugal-Tutkun et al., 2004). These data support the biological plausibility that inflammation and vascular dysregulation can influence retinal oxygenation, but they do not establish that the AZOOR pattern observed here is pathognomonic. A more conservative interpretation is that AZOOR may show a disease-associated metabolic profile compatible with localized inflammatory or vascular dysregulation, which requires prospective confirmation with lesion-mapped oximetry, fluorescein angiography, OCTA, and longitudinal functional testing. Because retinal oximetry was not consistently obtained during the acute symptomatic phase, the present findings should not be interpreted as evidence of acute inflammatory activity but rather as a disease-associated metabolic pattern observed during routine clinical follow-up.

### Treatment and limitations

Treatment interpretation remains limited. Some patients received systemic corticosteroids, antiviral therapy, antibiotics, or later immunomodulation. The patient with the longest diagnostic delay and no initial systemic corticosteroid therapy had the poorest visual outcome; however, causality cannot be inferred from this retrospective case series. The oximetry findings should therefore be interpreted as imaging biomarkers rather than as treatment-response markers.

This study has several limitations. First, AZOOR is rare and the sample size was small. Second, retinal oximetry was available only in four patients because the Oxymap system was introduced after several patients had already completed their clinical management. Consequently, retinal oximetry was unavailable for these earlier cases because the examination had not been performed rather than because of inadequate image quality or exclusion after image acquisition. Third, the oximetry data were summarized at eye/image level and were not prospectively standardized by lesion extent or perfusion territory. Fourth, both eyes from the same individual were included whenever available. Consequently, the effective number of independent observations was smaller than the number of analyzed eye/image measurements. Therefore, all statistical analyses should be regarded as exploratory and hypothesis-generating rather than confirmatory. Fifth, RP and control data came from a separate dataset and may differ in acquisition context. In addition, a standardized clinical severity classification was not available for the RP cohort, which limits disease-stage-specific interpretation of the comparison. Sixth, multiple exploratory comparisons were performed without correction. The oximetry findings should therefore be regarded as hypothesis-generating and suitable for planning prospective imaging studies rather than as definitive diagnostic thresholds.

Despite these limitations, the study demonstrates how retinal oximetry can be integrated into multimodal AZOOR assessment. For the Frontiers research topic on innovations in ophthalmic imaging, the key contribution is the addition of a metabolic vascular parameter to structural and electrophysiological retinal phenotyping. Future studies should combine A-SO_2_, V-SO_2_, and A-V SO_2_ with OCT layer segmentation, wide field FAF/NIR-R lesion mapping, OCTA, adaptive optics imaging, and longitudinal visual field, ffERG or mfERG outcomes.

## Conclusion

Multimodal imaging remains central to early recognition and monitoring of AZOOR. Retinal oximetry provides complementary metabolic information and, in this exploratory dataset, demonstrated lower A-SO_2_ and lower A-V SO_2_ in AZOOR compared with RP and healthy controls, while V-SO_2_ did not differ significantly. These results require prospective confirmation but support retinal oximetry as a candidate imaging biomarker in AZOOR and other outer retinal disorders.

## Data Availability

The data analyzed in this study is subject to the following licenses/restrictions: the anonymized data supporting the conclusions of this article may be made available upon reasonable request, subject to institutional and ethical restrictions. Requests to access these datasets should be directed to scott.tschuppert@h-och.ch.
